# Human-Risk-Aware Safe Path Planning Based on Reinforcement Learning for Autonomous Mobile Robots

**DOI:** 10.3390/s25237211

**Published:** 2025-11-26

**Authors:** Zhongjie Long, Xianbo Zhang, Jian Mi, Jun Wang

**Affiliations:** 1 College of Mechanical and Electrical Engineering, Key Laboratory of the Ministry of Education for Modern Measurement and Control Technology, Beijing Information Science & Technology University, Beijing 102206, China; zhongjielong@bistu.edu.cn (Z.L.); 2023020024@bistu.edu.cn (X.Z.); 2 Department of Transport Engineering, School of Civil Engineering and Transportation, Yangzhou University, Yangzhou 225127, China; 3 College of Information Science & Technology, Beijing University of Chemical Technology, Beijing 100029, China; wangjunrob@buct.edu.cn

**Keywords:** mobile robot, safe path planning, human-shared environments, uncertainties, stochastic risk evaluation

## Abstract

This paper addresses the challenge of safe path planning for mobile robots operating in human-shared environments, where human movements are inherently stochastic. To this end, we propose a reinforcement-learning-based path planning algorithm that accounts for human-related uncertainties at the planning level. The algorithm first employs a Markov decision process learner to explore the environment and generate multiple candidate paths. Second, to reduce computational redundancy, a path eliminator module filters out similar paths based on a proposed diversity metric, ensuring path diversity with minimal overhead. Simultaneously, a Monte Carlo-simulated human risk predictor is integrated into the decision-making unit to select the safest path among the candidates. This integrated algorithm enables robots to generate safe and efficient trajectories without the need for frequent re-planning, even in environments with stochastic human behavior. Simulation results demonstrate the effectiveness of the proposed method. In high-density settings, a 40×40 grid map with 10 humans, the proposed method reduces the average number of conflicts by −69.8%, −54.8%, and −73.4% compared with A*, MDP, and RRT methods, respectively. Meanwhile, it improves task success rates by 94.4%, 70.7%, and 118.75% relative to the same baseline methods.

## 1. Introduction

In recent decades, mobile robot technology has made significant advancements and has been widely applied in automated factories [1], warehouses [2,3], and logistics systems [4] for a variety of tasks, including inspection [5], pickup, and delivery [6]. Nowadays, the demand for mobile robots capable of collaborating safely and effectively with humans is rapidly increasing. Ensuring the safe and efficient operation in the presence of human uncertainty has thus become a critical challenge.

Path planning is a fundamental problem in robot control and navigation. It has been extensively studied in static environments with fully known domain knowledge, where it is typically formulated as the shortest path planning (SPP) problem [7], aiming to minimize travel distance or time. Classical algorithms such as Dijkstra’s algorithm [8,9,10] and A* [11,12,13] have been widely adopted to solve the SPP. In complex warehouse environments, real-world conditions often present dynamic and unpredictable challenges—such as sudden obstacles, human traffic, and changing operational demands—that significantly jeopardize mobile robot navigation and task execution. Given these risks, however, a previous study [14] indicated that online experimentation involving human participants is potentially unsafe, while developing a sufficiently accurate simulator often proves infeasible; These constraints make offline reinforcement learning (RL) a particularly promising alternative, as it enables learning to effectively influence sub-optimal human behaviors by synthesizing interaction strategies from recorded human–human demonstrations. Motivated by these insights, this paper focuses on planning a safe path at the planning level for mobile robots operating in environments characterized by uncertain human behavior.

In general, mobile robot path planning can be categorized into offline and online approaches. Offline path planning is typically employed in static environments with complete domain knowledge. A common example is warehouses, where automated guided vehicles (AGVs) operate using precomputed paths in structured settings. In contrast, online path planning is more flexible and suitable for dynamic and partially observable environments [15], where the robot makes real-time decisions based on sensor data or reciprocal velocity obstacle (RVO) [16,17]. It is widely considered one of the most effective methods for obstacle avoidance in dynamic settings [18], as it enables robots to respond adaptively to unexpected environmental changes. For instance, when a dynamic obstacle enters the robot’s workspace, algorithms such as those in  [19,20] can generate a collision-free local path to avoid both static and dynamic obstacles. However, frequent re-planning can reduce efficiency, especially in highly uncertain environments. It incurs high computational costs due to the need for continuous re-planning. Moreover, it is prone to being trapped in local optima, which can result in a lower task success rate.

In addition to the offline and online approaches mentioned above, safe path planning can also be achieved through non-learning approaches, such as cooperative decision making in shared spaces [21,22,23] and interaction-aware model predictive decision making [24]. These algorithms are commonly used to enable agents (e.g., autonomous vehicles, robots) to operate safely and efficiently in complex environments with other agents. They do not rely on learning policies from large datasets but instead perform online computations based on explicit physical models and behavioral rules. Therefore, these algorithms are characterized by strong interpretability and relatively high computational efficiency. However, their effectiveness heavily relies on the core assumption that all participants are rational and cooperative. In reality, there will always be non-cooperative or aggressive drivers or pedestrians, which can disrupt the entire collaborative model and lead to system failure. When there are more interacting agents, the complexity of the game increases exponentially (combinatorial explosion) and cannot be solved in real time. Also, simple rules cannot handle all corner cases.

RL has emerged as a promising approach due to its ability to model complex interactions, adapt to dynamic conditions, and improve performance over time through trial and error. RL has proven effective in decision making and learning optimal control policies [25,26]. Since the seminal work by Mnih et al. [27], numerous deep reinforcement learning (DRL) methods [28,29,30] have been developed for planning in dynamic environments. Despite these advances, RL still faces several limitations. For instance, when the environment is extremely large, the reward becomes sparse, inducing an increased training effort and making the overall learning process inefficient [31]. Another challenge is the over-fitting issue. A hierarchical deep learning (DL)-based control framework was proposed in [32] to plan optimal maneuver trajectories and guide mobile robots toward targets in uncertain environments. The proposed motion planning scheme was trained offline on a pre-generated dataset of optimized maneuver trajectories, enabling it to predict optimal motions in real time. In this way, the time-consuming online optimization process can be avoided. However, it is well known that neither RL nor DL methods can provide formal guarantees on the safety or optimality of the generated paths. Although RL-based methods have achieved remarkable success in various decision-making and control tasks, they still suffer from several limitations in global path planning when interacting with stochastically moving humans. The first limitation lies in the reward sparsity and instability, which severely hinder the learning process as informative feedback is only obtained upon reaching the goal or encountering collisions, leading to slow convergence and unstable policy updates. Another limitation arises from the high-dimensional and stochastic nature of human motion, which introduces significant uncertainty into the state transitions and makes it difficult for the agent to accurately capture the underlying environmental dynamics. Moreover, the exploration efficiency of RL algorithms is greatly reduced in such non-stationary environments, as the temporal variability of human trajectories renders previously collected experiences less reliable. To address these challenges, we propose a novel RL-based framework integrating probabilistic risk estimations for optimal global path planning in environments shared with stochastically moving humans.

The key challenge of path planning in dynamic environments is that uncertainties are hard to precisely predict. To handle uncertainties of moving obstacles or humans, one approach is to model and predict their trajectories [33,34]. Yet, separating the navigation problem into distinct prediction and planning stages can result in the well-known ‘freezing robot’ problem [35], as this approach still functions as a local, reactive planner rather than a truly global planning strategy. Conditional value at risk (CVaR) [36,37] has been applied in risk-aware path planning to manage uncertainty in dynamic environments. For example, Tan et al. [38] presented a risk-aware path planning method for specific battlefield terrains, thus allowing the autonomous system to ask a human teammate for help in reducing uncertainty and facilitating task progression. Chu et al. [39] incorporated CVaR into constraint formulation for moving obstacle avoidance, and the results showed the effectiveness of CVaR. Nevertheless, this method still follows a hierarchical framework, in which global path planning guides the robot toward its goal, while local planning is responsible for real-time obstacle avoidance. Moreover, CVaR constraints are hard to calculate.

Addressing potential collisions at the planning level has been shown to be an effective solution [40] and is considered more practical for real-world applications. A well-designed path planner must not only ensure safety in the presence of stochastic human behaviors but also maximize task success rates. This motivates the development of planning algorithms that can explicitly account for environmental uncertainty and human–robot collaboration at the planning level.

**Figure 1:**
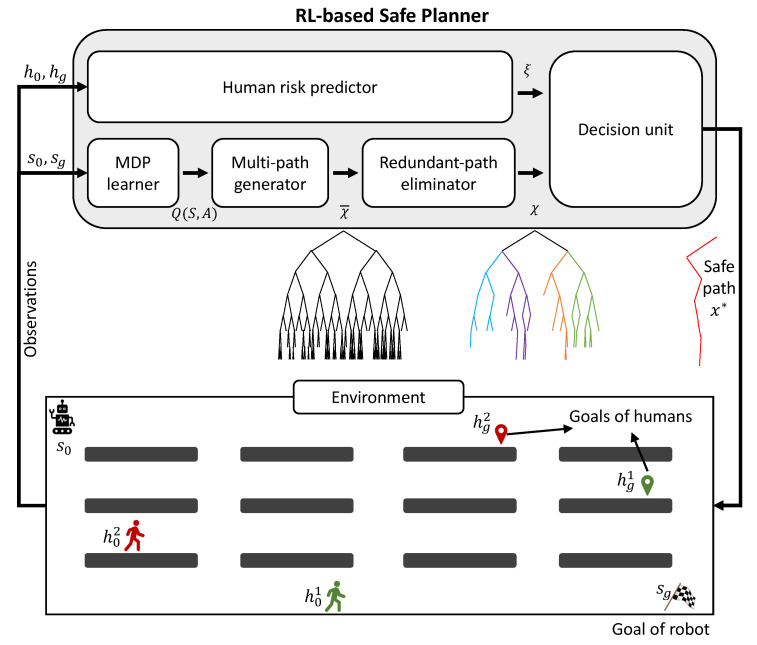
The entire architecture of the proposed safe planner. The environment shows a warehouse where a mobile works with humans. The gray rectangles are the obstacles such as shelves.

To generate a safe path for a mobile robot working with stochastically moving humans, we develop a reinforcement-learning-based safe path planning algorithm that solves collisions at the planning level. The overall architecture of the proposed safe planner is illustrated in Figure 1. Specifically, we first employ a Markov decision process (MDP) learner to generate multiple candidate paths, referred to as the *multi-path generator*. Paths that exhibit high similarity are filtered out by a *redundant-path eliminator* to ensure path diversity and reduce computational redundancy. We perform Monte Carlo simulations with the *human risk predictor* to estimate human motion risks. Finally, a *decision unit* determines an optimal path based on the predicted future risk. The contributions of this study are summarized as follows:

1. We introduce an RL-based multi-path generation method which adopts an optimal-first policy for finding multiple optimal/sub-optimal path candidates.
2. A novel redundant path eliminator, based on both distance metrics and cosine similarity, is developed to maintain path diversity while minimizing the path candidate set.
3. A human risk-based optimization mechanism is developed for obtaining an optimal safe path under human uncertainties where we run Monte Carlo simulations to build human risk constraints, and a safety-first policy is applied for path optimization.

The remainder of this paper is organized as follows: Section 2 formulates the problem formulation. Section 3.2 models the environment using an MDP. The proposed safe path planner is detailed in Section 3. Experimental comparisons and analysis are provided in Section 4, and Section 5 concludes the paper.

## 2. Problem Formulation

Typically, we consider an agent operating in an environment where humans move stochastically. The agent is required to complete a specific task in an assigned position. The agent operates in an environment shared with *K* humans, such as a warehouse environment. Each human has a corresponding goal position. The humans move with stochastic strategies in the environment and cannot be controlled, leading to uncertainties for the agent in reaching its goal. The agent may encounter a conflict with a human *k*(k∈{1,…,K}) at time step *t*. We formulate the path-finding problem as a discrete problem and model the environment as a grid world [41]. The bottom part of Figure 1 illustrates an agent operating alongside humans in a warehouse grid environment. Both the agent and the humans are assigned their own tasks, each requiring them to move to a specific goal position. The agent must reach its goal without colliding with any humans. It should be noted that humans move without considering the movements of the agent; therefore, the agent is responsible for avoiding collisions with them.

Let

- *S* be the state set of the agent; $s_{t}\in S$ denote the state of the agent at time step *t*; $s_{0}$ be the starting position; $s_{g}$ define the goal position;
- *H* be the state set of humans; $h_{t}=\left\{h_{t}^{1},h_{t}^{2},\ldots ,h_{t}^{k}\right\}\in H$ denote the state of *K* humans at time step *t*; $h_{g}=\left\{h_{g}^{1},h_{g}^{2},\ldots ,h_{g}^{k}\right\}\in H$ denote the state set of the goal corresponding to *K* humans;
- A={forward,backward,left,right,wait} define the action set of the agent and humans; given a time step *t*, the action set of the agent at state $s_{t}$ is denoted as $A(s_{t})\subseteq A$; the action set that the human *k* can take at $h_{t}^{k}$ is $A(h_{t}^{k})\subseteq A$.

Therefore, a *solution* is a path for the agent, written as $\pi =\{s_{0},s_{1},\ldots ,s_{g}\}$, recording the trajectories from the start state $s_{0}$ to a goal $s_{g}$. The agent performs its task within a time-step budget *T*. A conflict occurs when the agent and a human *k* occupy the same position or traverse the same edge, that is,

- A vertex conflict occurs when $s_{t}=h_{t}^{k}$, and
- An edge conflict occurs when $s_{t}=h_{t+1}^{k}\wedge s_{t+1}=h_{t}^{k}$.

A task is considered successful if and only if an agent reaches its goal without collisions and within the time-step budget *T*. Otherwise, the task is considered a failure.

The environmental uncertainties primarily arise from the stochastic movements of humans. Although the goals of the humans are known, their paths remain uncertain. In this study, our aim is to find a safe solution among multiple paths to ensure that the agent reaches its goal without collisions. We place greater emphasis on the safety of the solution than the shortest path.

## 3. Proposed Safe Planner

### 3.1. Architecture

We propose an RL-based safe path planner to find an optimal safe path for an agent in a shared workspace with humans. As illustrated in Figure 1, the architecture comprises five core modules:

1. MDP learner: Explores the environment and gain experience, Q-table.
2. Multi-path generator: Multiple optimal or sub-optimal paths are generated based on the MDP learner, forming a set of candidate paths that serves as the initial solution set.
3. Redundant-path eliminator: The path candidate set is refined using our proposed path diversity metric.
4. Human risk predictor: Accounting for human uncertainties, a conditional risk set is generated by integrating probabilistic modeling with spatiotemporal trajectory prediction.
5. Decision unit: Finding the optimal safe path from the refined set using quantitative human risk assessment.

The detailed processing methodology will be presented in the following section.

### 3.2. Environment Modeling: Markov Decision Process

The agent works in a 2D grid ($D_{x}\times D_{y}$) world, denoted by G=<V,E>, where *V* represents the set of vertices and *E* the set of edges. We use MDP to model the motions of the agent as $M=(S,s_{0},s_{g},A,P,R,\gamma )$ where

- *S* is the state set of the agent, $\forall s\in S,s=(v_{x},v_{y})$, where $v_{x}$ and $v_{y}$ denote the coordinates of vertex *v* in the grid world; $s_{0}\in S$ is the initial state of the agent; $s_{g}\in S$ is the goal state;
- *A* is the finite set of actions that the agent can take;
- *P* is the state transition probability: S×A×S→[0,1];
- *R* is the reward function: $R(s,a,s^{\prime })\to R$, where *s* and $s^{\prime }\in S,a\in A$;
- γ is the discount factor: γ∈[0,1].

Taking an action $a_{t}\in A\left(s_{t}\right)$ at any time step *t*, the agent moves to a state $s_{t+1}$ from $s_{t}$ with a probability $p(s_{t+1}|s_{t},a_{t})$, where $\Sigma_{a_{t}\in A\left(s_{t}\right)}p\left(s_{t+1}|s_{t},a_{t}\right)=1$. The agent will obtain a reward $R(s_{t},a_{t},s_{t+1})$. The discount factor γ is used to balance immediate and future rewards.

Q-learning is used to learn an optimal policy with the Bellman equation. The learned Q-values are recorded in the Q-Table, and each entry (Q-value) corresponds to a state–action pair. The Q-values are updated by the following equation:

(1) $$\begin{array}{cc} Q(s,a)\leftarrow & Q(s,a)+\\ & \alpha \left(R\left(s,a\right)+\gamma \underset{a^{\prime }\in A\left(s^{\prime }\right)}{max}\;Q\left(s^{\prime },a^{\prime }\right)-Q\left(s,a\right)\right), \end{array}$$

where α is the learning rate.

### 3.3. MDP Learner and Multi-Path Generator

Unlike the classic MDP approach that learns an optimal policy, the MDP learner in this paper enhances the exploration of the workspace and gains a comprehensive Q-table, which means it covers the workspace as much as possible. Hence, the parameter ϵ in ϵ-greedy is set to a larger value than in the classic MDP for enhancing environment exploration.

**Figure 2:**
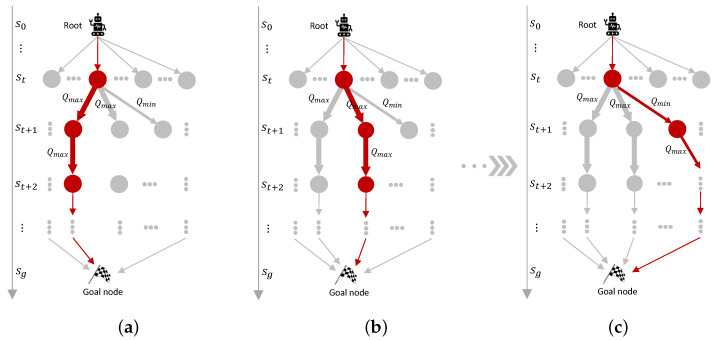
Schematic diagram of the proposed multi-path search process. (**a**) Optimal path #1. (**b**) Optimal path #2. (**c**) Optimal path #3.

Based on the learned Q-table Q(S,A), we construct a multi-path generator to obtain multiple optimal/sub-optimal paths. An optimal path $x=\{s_{0},s_{1},\ldots ,s_{t},\ldots ,s_{T}\}$ can be easily obtained based on the learned Q(S,A) with a greedy policy, as shown in Figure 2a. *x* is a base path to generate multiple optimal/sub-optimal paths.

**Definition 1.** 
*$\forall s_{t}\in x$, if $|A(s_{t})|\geq 4$, we say $s_{t}$ is a potential anchor and let P_list be the set of all potential anchors.*

**Definition 2.** 
*$\forall s_{t}\in P\_list$, if it has more than one action that has the maximum $Q(s_{t},a_{t})$, we say $s_{t}$ is a branch anchor and let B_list contain all branch anchors. Let $A_{m}\left(s_{t}\right)$ be the optimal action set at $s_{t}$ and $A_{m}\left(s_{t}\right)\subseteq A\left(s_{t}\right)$.*

Hereafter, we describe how multiple paths are generated in detail. If B_list≠∅, we select a branch anchor $s_{t}$ using a random policy. From the chosen branch anchor $s_{t}$, an optimal action $a\in A_{m}\left(s_{t}\right)$ is randomly selected and another optimal path $x^{\prime }$ is obtained, shown as Figure 2b. Then, the corresponding optimal action *a* is removed from $A_{m}\left(s_{t}\right)$. If $A_{m}\left(s_{t}\right)$ becomes empty, the branch anchor $s_{t}$ is removed from B_list. In addition, P_list and B_list are updated by the newly generated path $x^{\prime }$. Similarly, we can generate multiple optimal paths until B_list becomes empty. All optimal paths are recorded in an optimal path set $X_{opt}$.

When B_list=∅ and P_list≠∅, the generator begins to search sub-optimal paths. For each $x=\left\{s_{0},\ldots ,s_{t},s_{t+1},\ldots ,s_{g}\right\}\in X_{opt}$, we first find out the potential anchors in path *x*. For each potential anchor $s_{t}$, it satisfies $s_{t}\in x\wedge P\_list$. We randomly choose a potential anchor and generate a new path $x_{new}=\left\{s_{0},\ldots ,s_{t},s_{t+1}^{\prime },\ldots ,s_{g}\right\}$ by visiting another branch from $s_{t}$ based on the learned Q(S,A). Note that in the optimal path *x*, $Q(s_{t},a_{t})$ is the largest and the state transition is $\left(s_{t},a_{t},s_{t+1}\right)$. In the newly generated path $x_{new}$, $Q(s_{t},a_{t}^{\prime })$ is the second largest and the state transition is $\left(s_{t},a_{t}^{\prime },s_{t+1}^{\prime }\right)$ where $a_{t}^{\prime }\neq a_{t}$. *x* and $x_{new}$ share the same part, $\left\{s_{0},\ldots ,s_{t}\right\}$. The differences are in the path from $s_{t}$ to $s_{g}$. The sub-optimal paths are generated similarly. The generated paths are recorded in $X_{s}$.

The multiple paths are finally generated and $X=X_{opt}\cup X_{s}$. Note that ∀x∈X, its length |x|≤T. The multi-path generator stops searching if |X|>N where *N* is the desired path number. Another termination when P_list = ∅ where the multi-path generator returns X with path number less than *N*. The whole process of the multi-path generator is presented in Algorithm 1. If the multi-path generator fails, $\bar{\chi }=\emptyset$, the robot stays and report no available path candidates. The time computational complexity is O(N|B_list||A|+N) and the space computational complexity is O(N).

**Algorithm 1:** Multi-path generator

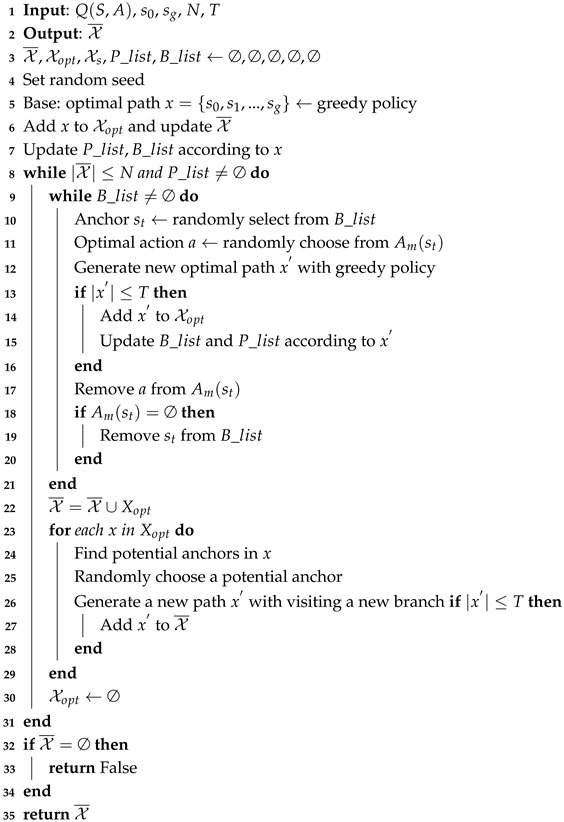



### 3.4. Redundant-Path Eliminator

To ensure the diversity of the generated paths, this module is designed to eliminate highly similar paths from set $\bar{X}$. The operation consists of two steps: (1) obtaining baseline paths from set $\bar{X}$, followed by (2) deriving a multi-path solution set X through an iterative method based on these baseline paths. Based on the starting point $s_{0}$ and the goal $s_{g}$ shown in Figure 1, two edge-following paths exhibiting maximal diversity are selected as the baseline paths. The whole process of path elimination is presented in Algorithm 2. The time computational complexity is $O(N^{2})$ and the space computational complexity is $O(N^{2})$.

**Algorithm 2:** Path elimination algorithm.

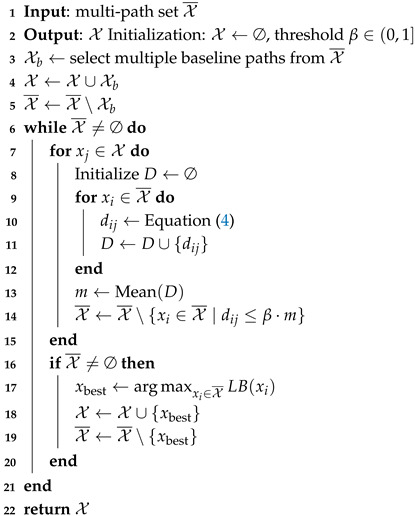



In this study, the elimination of similar paths was determined by a diversity metric evaluated through both distance measures and cosine similarity. Given any two paths $x_{i}$ and $x_{j}$, the distance at timestep *t* is defined as

(2) $$d_{t}=\frac{2}{D_{x}+D_{y}}Manh\left(s_{t}^{i},s_{t}^{j}\right),$$

where $D_{x},D_{y}$ define the map size, and Manh(·) denotes the Manhattan distance between states $s_{t}^{i}$ and $s_{t}^{j}$.

The cosine similarity at timestep *t* is calculated by

(3) $$c_{t}=1-cos\left({\vec{s}}_{t}^{i},{\vec{s}}_{t}^{j}\right),$$

where vectors ${\vec{s}}_{t}^{i}=s_{t}^{i}-s_{t_{0}}$ and ${\vec{s}}_{t}^{j}=s_{t}^{j}-s_{t_{0}}$.

Distance $d_{t}\in \left[0,2\right]$ is employed to quantify spatial positional deviations, while $c_{t}\in \left[0,2\right]$ is utilized to assess the diversity of motion direction. A smaller $d_{t}$ indicates closer proximity between $s_{t}^{i}$ and $s_{t}^{j}$, while a larger $c_{t}$ signifies a greater included angle between $\vec{s_{t}^{i}}$ and $\vec{s_{t}^{j}}$.

Based on Equations (2) and (3), we define the diversity metric of any two paths as

(4) $$Div\left(x_{i},x_{j}\right)=\frac{\sum_{t=1}^{max(T_{x_{i}},T_{x_{j}})}min\left(d_{t},c_{t}\right)}{max\left(L_{x_{i}},L_{x_{j}}\right)},$$

where $T_{x}$ indicates the ended timestep of path *x*, min(·) and max(·) are the minimum and the maximum functions, and $L_{x}$ indicates the length of path *x*. Consequently, a higher value $Div\left(x_{i},x_{j}\right)$ indicates a greater diversity between the paths $x_{i}$ and $x_{j}$, while a lower value denotes a greater similarity.

Additionally, the low bound function of the diversity is defined as

(5) $$LB\left(x_{i}\right)=min_{x_{j}\in X}Div\left(x_{i},x_{j}\right).$$

It should be noted that employing the min function (instead of max) in this formula ensures that the chosen path exhibits substantial divergence from every baseline path.

### 3.5. Human Risk Predictor

**Figure 3:**
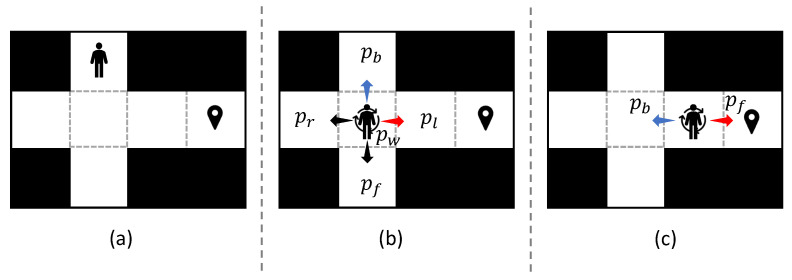
Stochastic model of humans. (**a**) Timestep (t−1). (**b**) Timestep (*t*). A human moves to his/her goal or take a “wait” action ($p_{w}$) with a random policy. The black area indicate obstacles. The red arrow represents the prioritized action, while the blue arrow depicts the revisitation action of the previous step. (**c**) Timestep (t+1).

The human risk assessment in this study is calculated using stochastic simulations within a 2D grid world. Figure 3b shows that a human takes an action *a*(a∈A) with a probability $p_{a}$, and $p_{a}\in \left\{p_{f},p_{b},p_{l},p_{r},p_{w}\right\}$ is the probability set with respect to action set *A*. $p_{a}$ is unknown as the human is not controlled by the system and his movement is self-determined.

To better emulate natural human movement, we assume that

- Conflicts will not occur between any two humans;
- A human prefers to move toward his goal.

Based on the above rules, we define the motion model of the humans as follows. Figure 3 illustrates a stochastic motion model of humans. A human *k* takes an action $a_{t}$ at $h_{t}^{k}$ and moves to next position, written as $\left(h_{t}^{k},a_{t},h_{t+1}^{k}\right)$. $\forall a_{t}\in A\left(h_{t}^{k}\right)$,

(6) $$a^{*}\leftarrow \arg\min_{a\in A(h_{t}^{k})}Manh\left(h_{t+1}^{k},h_{g}^{k}\right),$$

where Manh(·) calculates the Manhattan distance from $h_{t+1}^{k}$ to $h_{g}^{k}$.

Let *C* denote the prioritized action set, $C\subseteq A(h_{t}^{k})$, and it contains all actions satisfying Equation (6). Prioritized actions are assigned higher conditional probabilities to facilitate goal-directed human movement. On the basis of this, human actions are selected according to the following conditional probability distribution:

(7) $$P\left(a_{t}|h_{t}^{k}\right)=\left\{\begin{array}{cc} \frac{1-\zeta \left(|A(h_{t}^{k})|-|C|\right)}{\left|C\right|}, & a_{t}\in C\\ \;\zeta , & \mathrm{otherwise} \end{array}\right.$$

where |·| indicates the number of actions, $\sum_{a_{t}\in A\left(h_{t}^{k}\right)}p_{a_{t}}=1$, ζ is a probability parameter.

For a human *k*, let $h_{0}^{k}$ be the observed state at time step t=0, where $h_{0}^{k}=v$ means that a vertex v=(x,y) (vertex index x,y∈N) is occupied. We run stochastic simulations from the observed state $h_{0}^{k}$ to predict a human’s paths. The conditional probability of a vertex *v* occupied by a human *k* at time step t(0<t≤T) is computed as follows:

(8) $$p_{k,t}\left(v|h_{0}^{k}\right)=\frac{N_{v}}{N_{s}},$$

where $N_{s}$ denotes the number of simulation, and $N_{v}$ indicates the visited times of the vertex *v* at time step *t*. Consequently, the risk value of vertex *v* that is occupied by *K* humans is defined by

(9) $$R\left(v,t\right)=\sum_{k=1}^{K}p_{k,t}\left(v|h_{0}^{k}\right).$$

Finally, we obtain the human risk set $\xi =\{\xi_{1},\xi_{2},\ldots ,\xi_{T}\}$ for t∈(0,T]. We can easily determine the risk of each vertex *v* at any time step *t* from $\xi_{t}$. Note that the observed human positions at the current time step *t* is the only known point. We predict the future positions of humans by simulations. If we perform sufficient simulations, the accuracy of risk distribution can be improved. However, if only limited simulations are conducted, the accuracy of risk distribution would be low.

### 3.6. Decision Unit

To generate a safe path, we develop a safe path-finding method that performs a best-first search. The core idea is to take human risks into consideration. That is, the total risk is not only determined by the location *v*, but it is also affected by the future risk. Given a deterministic path $x_{i}$, the total risk is calculated by

(10) $$\begin{array}{cc} Risk\left(x_{i}\right) & =V_{r}\left(v\right)+E_{r}\left(v\right)\\ \; & =\sum_{t=0}^{T_{x_{i}}-1}R\left(v,t\right)+\sum_{t=0}^{T_{x_{i}}-1}\left(R\left(v,t+1\right)\times R\left(v^{\prime },t\right)\right), \end{array}$$

where $V_{r}\left(v\right)$ is the vertex conflict risk at location *v*, and $E_{r}\left(v\right)$ is the edge conflict risk from the current location *v* to the next location $v^{\prime }$ whose time step is t+1. Given that $\xi_{t}$ represents the human risk set at time step *t*, we can rewrite Equation (10) as

(11) $$Risk\left(x_{i}\right)=\sum_{t=0}^{T_{x_{i}}-1}\xi_{t}\left(s_{t}\right)+\sum_{t=0}^{T_{x_{i}}-1}\left(\xi_{t}\left(s_{t+1}\right)\times \xi_{t+1}\left(s_{t}\right)\right).$$

Consequently, the problem of finding the best-safe path becomes that of finding a path $x^{*}$ whose risk is the minimum among set X:

(12) $$x^{*}\leftarrow argmin_{x\in X}Risk\left(x\right).$$

The process of the decision unit is presented in Algorithm 3. The time computational complexity is O(|χ|T) and the space computational complexity is O(|χ|).

**Algorithm 3:** Decision unit.

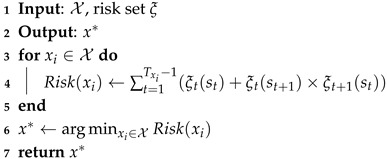



## 4. Simulations and Discussion

Inspired by real-world autonomous warehouse applications, recent studies on multi-agent path-finding [41] have conducted experiments using grid-based environments designed to mimic warehouse layouts, particularly featuring long corridors [42,43]. To evaluate the proposed methods, we carry out simulations under various scenarios and compare the results with those obtained from A*, MDP, RRT (Rapidly-exploring Random Trees) and DQN (Deep Q-networks). The simulations were conducted on a system with an Intel Core i9-10900 CPU (2.80 GHz, Intel, Santa Clara, CA, USA) and 32 GB memory. For all methods in the verification, we use the same reward definitions:

- Goal reward $r_{g}=10$;
- Step penalty $r_{s}=-0.1$;
- Conflict penalty $r_{c}=-2$.

Once the agent completes one task, it receives a reward for the achievement of the goal $r_{g}$. Meanwhile, the agent receives a step penalty $r_{s}$ for each step, also called step cost. As the environment is shared with humans, the agent is penalized with $r_{c}$ when it collides with a human.

We evaluate the performance of the proposed methods from the following aspects: average conflict number, conflict distribution, task success rate, and reward. In each scenario, we simulate 100 times and calculate the average value for evaluation. The other parameters are as follows:

- Learning rate α=0.7;
- Discount factor γ=0.9;
- Exploration rate ϵ=0.7;
- Number of episode = 4000;
- Human move simulation = 2000;
- Threshold (Algorithm 2) β=0.2;
- Probability parameter ζ∈[0,0.2).

**Figure 4:**
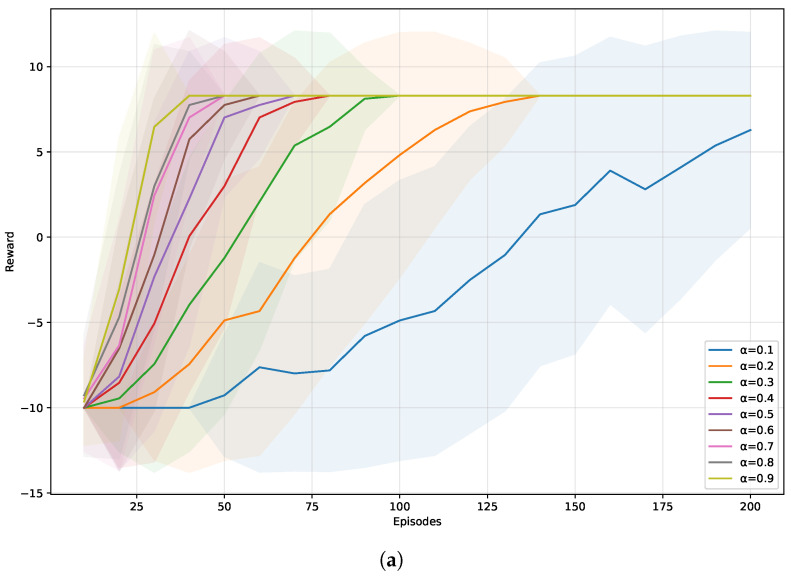

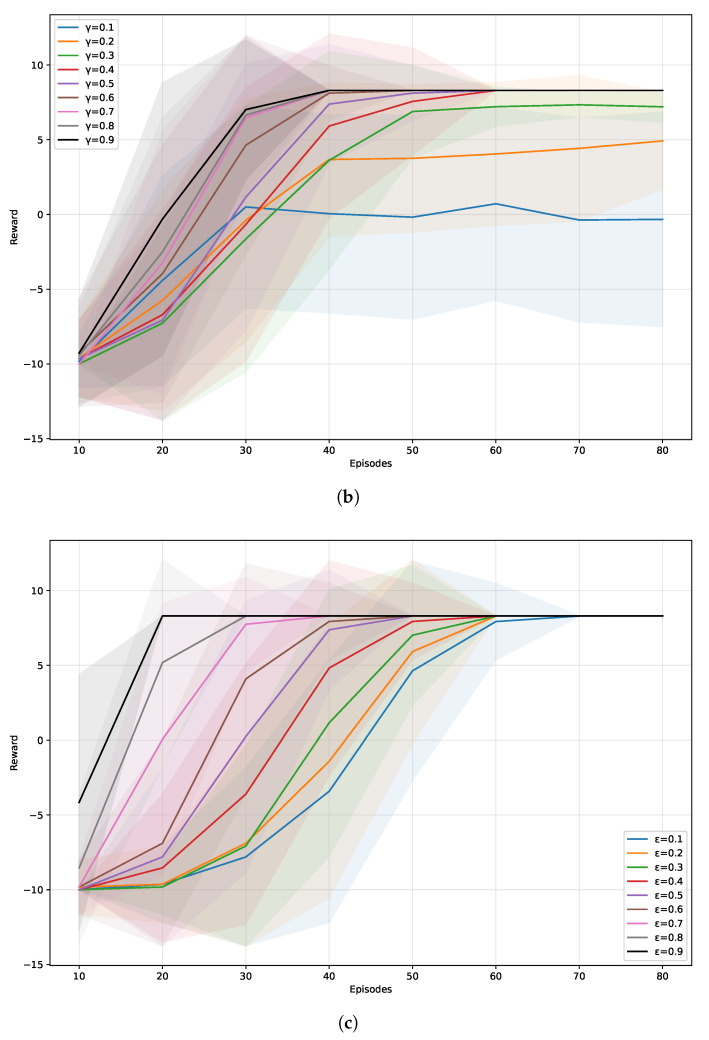
Parameter investigation: (**a**) Learning rate α. (**b**) Discount factor γ. (**c**) Exploration rate ϵ.

Figure 4 illustrates how different parameters influence the learning process. Figure 4a investigates the effect of α. As expected, α affects the learning speed. Table 1 shows the average results of conflict number, task success rate, and reward with 100 simulations. When α=0.7, the average conflict number is the least and the task success rate is high as 0.97. Thus, we set α=0.7. Figure 4b shows the effect of γ. As we expect the algorithm to place more emphasis on future rewards, we directly set γ=0.9, the same as in [40]. Table 2 presents the average results under different values of γ. It also shows that γ=0.9 is a good choice with fewer conflicts and higher task success rate. For the ϵ, we do not set the common value 0.2 [26,40] as our purpose is to enhance the exploration of the environment. However, how large ϵ should be is difficult to determine. As Figure 4c shows, they finally converged at the same reward and they did not vary much in the average conflict number and task success rate, as shown in Table 3. Finally, we set it manually as 0.7, which is not too large or too small.

From Table 1, Table 2 and Table 3, it can be clearly observed that the parameters α, γ, and ϵ have only a minor influence on the conflict number, task success rate, and path reward, which demonstrates that the proposed method is relatively insensitive to these parameters. This is mainly because the proposed method generates multiple path candidates over the entire map, which greatly improves the robustness and stability of the algorithm against stochastic human movements.

**Figure 5:**
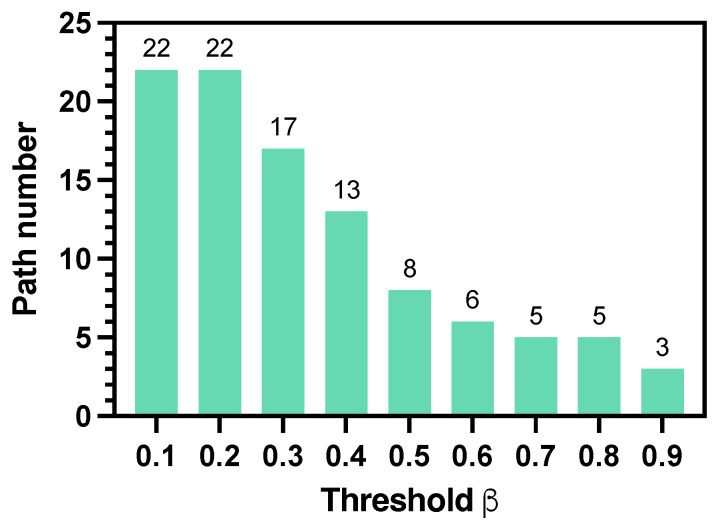
Path number of χ after elimination from $\bar{\chi }$ ($|\bar{\chi }|=136$) with different threshold β.

Figure 5 shows how the threshold β affects the elimination of redundant paths. We obtained $|\bar{\chi }|=136$ paths with the multi-path generator in a 10×10 grid environment. When β increases, more paths are eliminated. As Figure 5 shows, β=0.2 is a critical point where more paths are removed and |χ| changes sharply. In this paper, we keep a conservative strategy and choose to reserve more paths to enhance the robustness to human uncertainties and set β=0.2. As for the parameters in the human motion model, we set ζ∈[0,0.2). If the human moves according to a uniform distribution over all possible actions, the probability of each action would be 0.2. However, under the assumption that a human tends to move toward the goal, the probability of any action outside the prioritized action set should be smaller than 0.2. Therefore, we set ζ within this range to control the degree of stochasticity in human motion while maintaining goal-directed behavior.

For MDP and DQN methods, the robot receives a conflict penalty when it collides with a human during the exploration. Note that only the robot could be rewarded or penalized. A human never receives a reward and penalty as they are the uncertainties of the environment. For the A* method, we add a conflict cost along its step cost while the RRT also performs with a cost function for human avoidance.

### 4.1. Scenario 1: Verification on a 10×10 Grid World Environment

Scenario 1 is simulated in a 10×10 grid world. The agent starts from $s_{0}$ = (0, 0), and $s_{g}$ = (9, 9) is its goal position. A human moves stochastically from position $h_{0}^{1}$ = (9, 0) to his/her goal position $h_{g}^{1}$ = (0, 9). The time budget T=20. In this paper, the agent starts from the upper-left corner, and its goal is located at the lower-right corner, while the human starts from the upper-right and moves toward the lower-left. This cross configuration increases the likelihood of collisions between the agent and the human along any potential path. In addition, the human start and goal positions are fixed so that all 100 simulations are conducted under the same configuration, eliminating the randomness introduced by different human position settings. This ensures a more reliable evaluation of the proposed algorithm’s performance.

**Figure 6:**
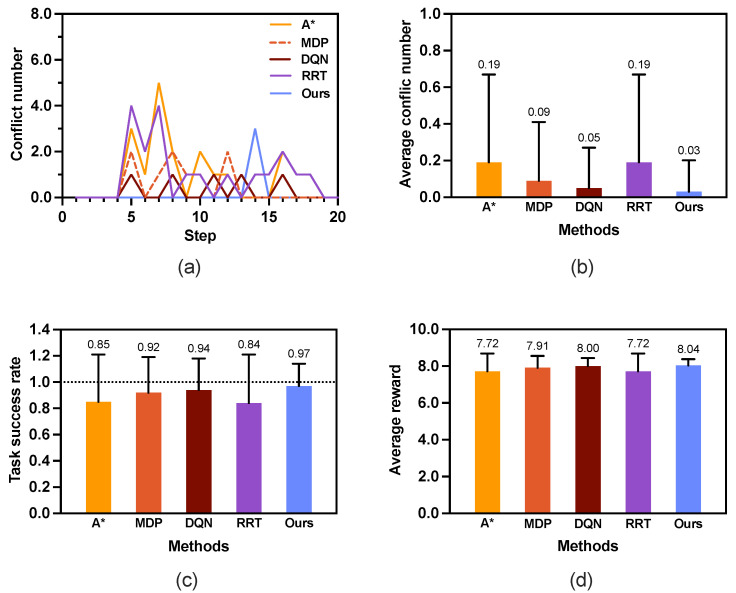
Results of scenario 1: a 10 × 10 grid map with a single human agent (K=1). The numerical value above each bar represents the mean performance obtained from 100 rounds of simulation. (**a**) Conflict distribution. (**b**) Average conflict number. (**c**) Average task success rate. (**d**) Average reward.

Figure 6a shows the conflict distribution that records the time step at which conflicts occur. In A* and MDP, the first conflict occurs at the same time step t=5. In contrast, the first conflict occurring in our proposed method is much farther behind than those in the other methods, which is at time step t=14. Figure 6b depicts the average conflict number in 100 rounds of simulations. The A* method has the highest average conflict numbers (0.19 ± 0.48) compared with the other methods. The proposed method has the least average conflict number, 0.03 ± 0.17, which is much less than the those of the other two methods.

The simulation results of scenario 1 indicate that our proposed safe planner not only reduces the conflict number but also delays the time at which a conflict occurs. Notably, the proposed method demonstrates superior performance, achieving a success rate of 0.97 ± 0.17, which is significantly higher than the other methods (see Figure 6c). Moreover, as shown in Figure 6d, our method achieves a higher reward of the generated path than the other two methods.

**Figure 7:**
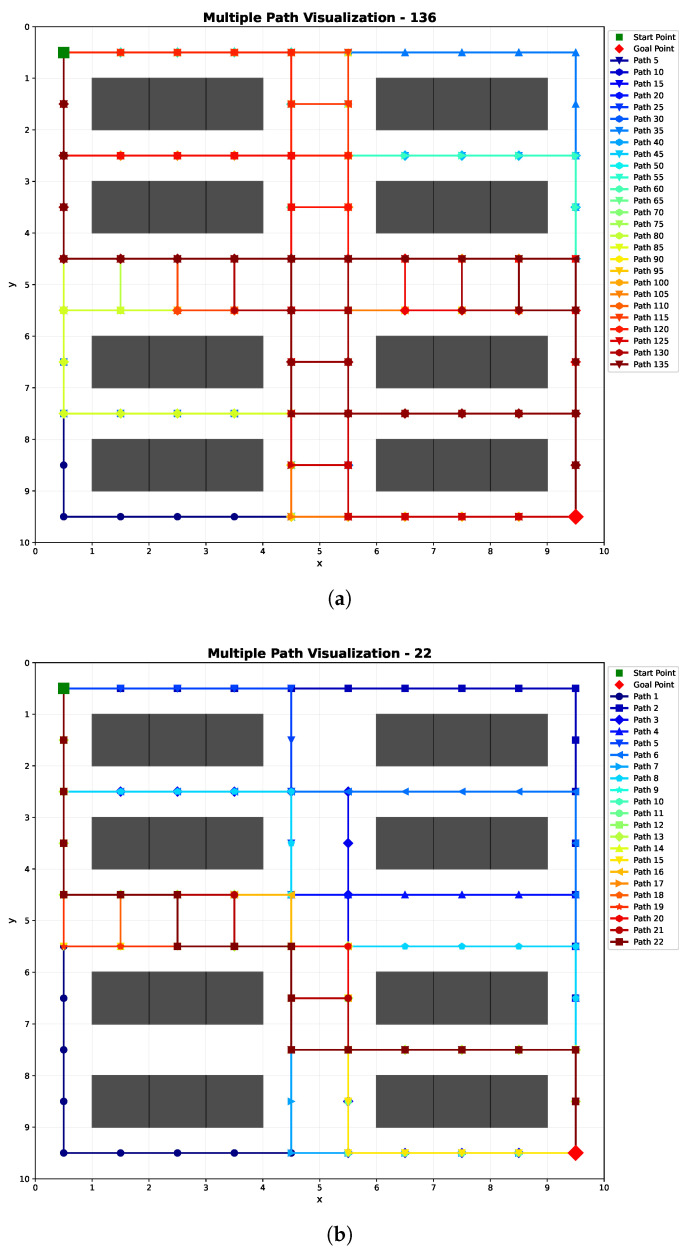

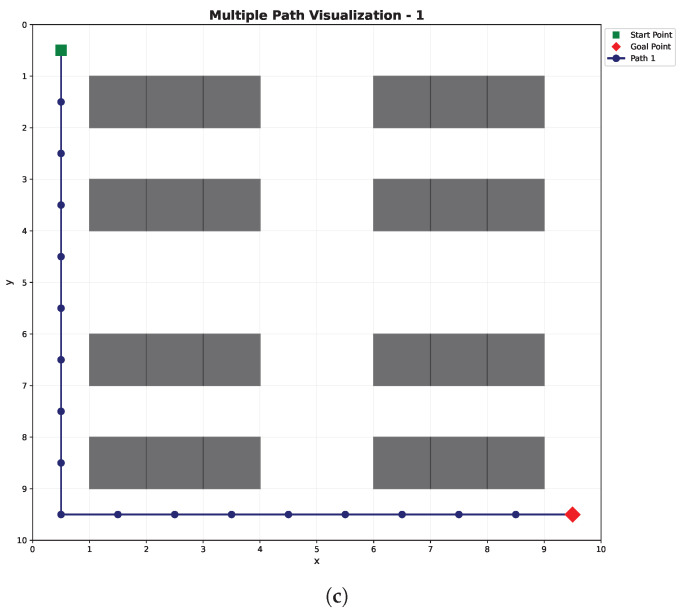
Illustrative examples of path generation on a 10×10 grid map. This figure shows the paths generated by our proposed method. (**a**) Diverse path set $\bar{X}$: The complete set of 136 generated paths. For clarity, the legend displays every fifth path. (**b**) Redundant path set X: A subset of 22 distinct paths obtained by removing highly similar ones from $\bar{X}$. (**c**) Safety-optimized path $x^{*}$: The ultimate path selected based on human risk assessment.

**Figure 8:**
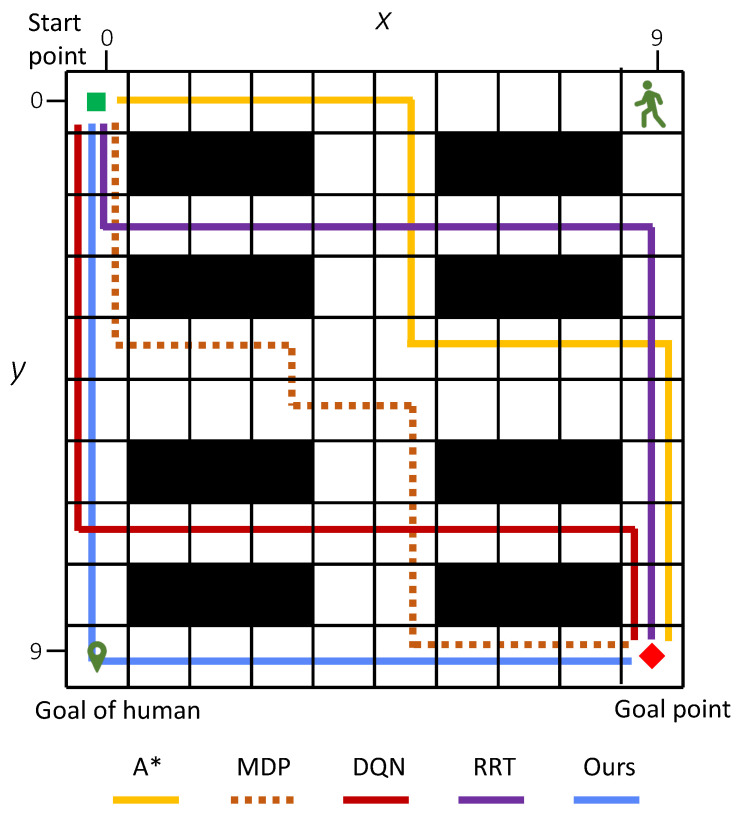
The generated paths in a 10×10 grid map environment with one human. Black areas represent obstacles.

Considering the average number of conflicts, task success rate, and path reward, the simulation results clearly demonstrate the effectiveness of our method. Figure 7 shows an example of an optimal path generated. Figure 7a illustrates the generated 136 paths, $\bar{\chi }$. Figure 7b shows the 22 diverse paths χ after path elimination where path number reduces by about 83.8%. Figure 7c shows the safety-optimized path $x^{*}$. An example of the generated paths for a 10×10 map of different methods is illustrated in Figure 8.

### 4.2. Scenario 2: Investigation on Different Map Size

In scenario 2, we investigate the performances of the proposed algorithm on different map size, 20×20 and 40×40. Only one human is considered in this scenario. The budgets are set as T=40 and T=80, respectively.

**Figure 9:**
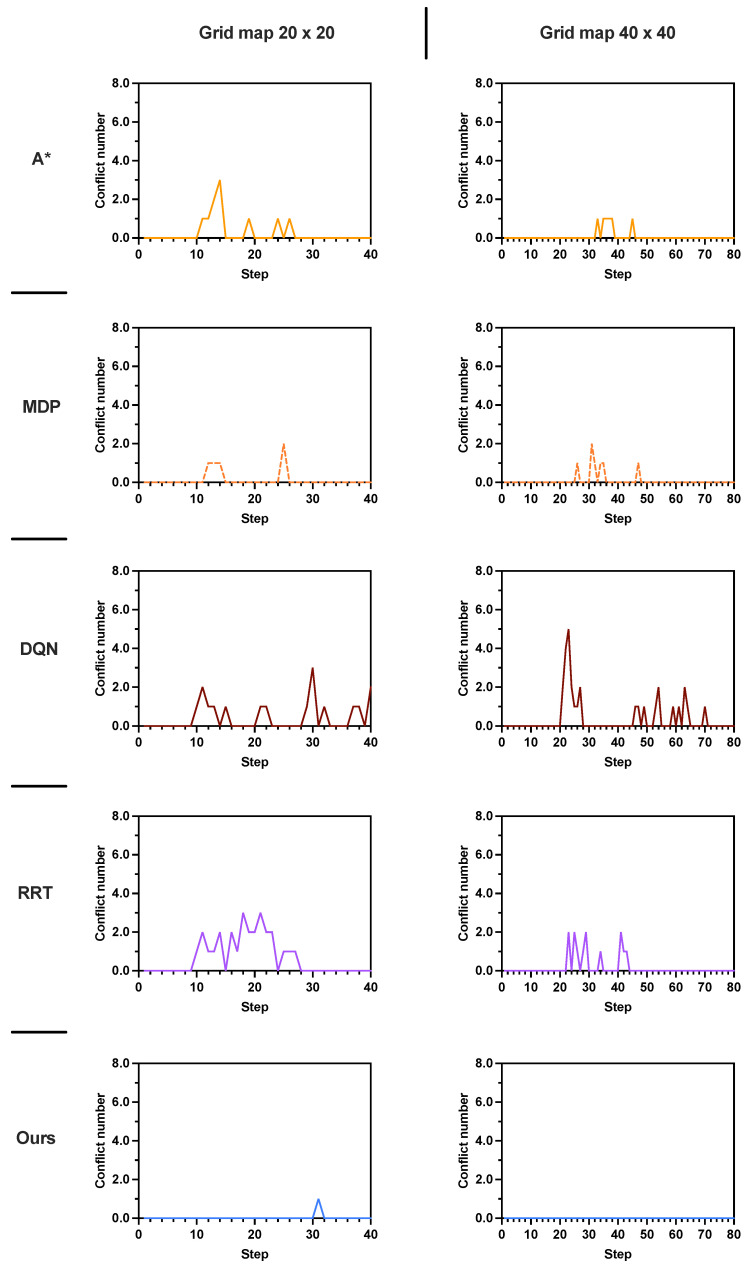
A comparison of conflict distributions in scenario 2 for the 20×20 and 40×40 grid maps (K=1). The conflict number represents the mean performance obtained from 100 rounds of simulation.

The simulation results are presented in Table 4 and Figure 9. As shown in Table 4, across all map sizes, the proposed method outperforms A* and MDP by achieving the lowest number of conflicts, the highest task success rate and the highest reward. Comparing the results across different map sizes 10×10, 20×20, and 40×40, we observe that the average number of conflicts decreases and the task success rate increases for A* and our proposed methods. This trend is primarily attributed to the fact that larger map sizes reduce the impact of uncertainties introduced by stochastic human movements. When the map size increases to 40×40 with sparse human density, the proposed method achieves an average task success rate of 1.0, with zero conflicts observed over 100 simulations. The conflict distributions are shown in Figure 9 (right).

In terms of average reward, the differences among the methods are marginal. Our proposed method achieves the highest average reward for all maps with minimal standard deviation, indicating reliable and consistent performance. All simulation results show that our method works well in large maps.

### 4.3. Scenario 3: Investigation on Increasing Human Numbers

In this scenario, we investigate the performance of the proposed method on a more complex environment by increasing the numbers of humans *K*. The simulation environment is a 40×40 grid world involving 2∼10 humans. The agent starts from $s_{0}$ = (0, 0), and $s_{g}$ = (39, 39) is its goal position. The humans’ starting point and target location are also randomized where $h_{0}^{1}$ = (30, 39), $h_{0}^{2}$ = (39, 0), $h_{0}^{3}$ = (4, 2), $h_{0}^{4}$ = (1, 37), $h_{0}^{5}$ = (29, 31), $h_{0}^{6}$ = (2, 33), $h_{0}^{7}$ = (14, 4), $h_{0}^{8}$ = (21, 8), $h_{0}^{9}$ = (30, 10), $h_{0}^{10}$ = (33, 12); their goal positions are $h_{g}^{1}$ = (6, 39), $h_{g}^{2}$ = (0, 39), $h_{g}^{3}$ = (35, 35), $h_{g}^{4}$ = (32, 6), $h_{g}^{5}$ = (6, 12), $h_{g}^{6}$ = (30, 2), $h_{g}^{7}$ = (38, 29), $h_{g}^{8}$ = (5, 27), $h_{g}^{9}$ = (6, 25), and $h_{g}^{10}$ = (17, 35), respectively. The budget is configured as T=80.

**Figure 10:**
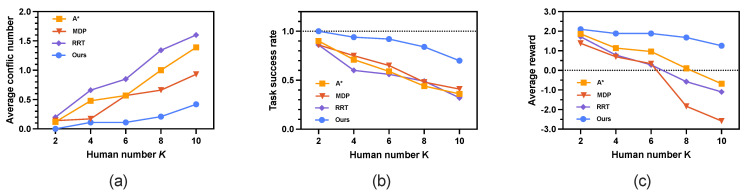
Simulation results on increasing human number *K*. The results represent the mean performance obtained from 100 rounds of simulation. (**a**) Average conflict number. (**b**) Average task success rate. (**c**) Average reward.

The simulation results evaluated by three key metrics are shown in Figure 10 and summarized in detail in Table 5. As shown in Figure 10a, the average conflict number increases for all methods as the number of humans *K* grows. The proposed method consistently outperforms the other three approaches, maintaining the lowest average conflict number. Even under high human density (K=10), our method effectively reduces conflicts, achieving an average conflict number of 0.42, which is 3∼4 times lower than A* (1.39) and RRT (1.60). As illustrated in Figure 10b, the proposed method also achieves the highest average task success rate across all values of K=2∼10. Furthermore, it yields the highest average reward (Figure 10c) as the number of humans increases. With increasing *K*, environmental uncertainty rises significantly, making it more challenging to plan safe paths without re-planning. The simulation results demonstrate that our method is more robust to such uncertainties compared to the other three approaches.

**Figure 11:**
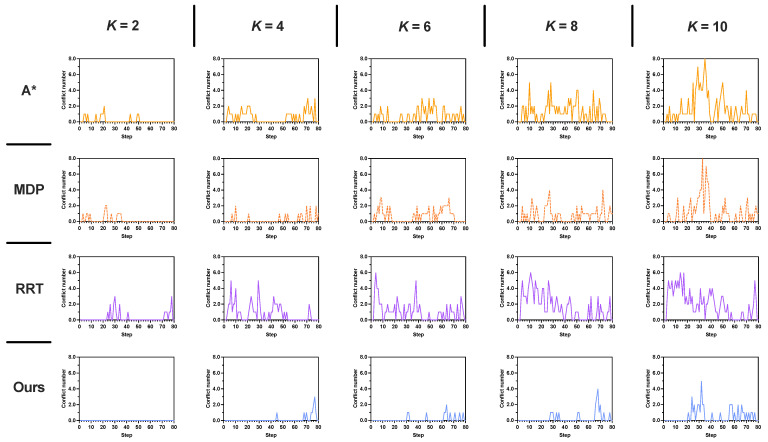
Conflict distribution of a 40×40 grid map with human number *K* = 4∼10. The conflict number represents the mean performance obtained from 100 rounds of simulation.

From human number K=4, the results of our method differ from the other three methods. For average conflict numbers, our approach maintains the lowest averages (0.11∼0.53) with smaller standard deviations compared with A* (0.48∼1.21), MDP (0.17∼0.38), and RRT (0.66∼1.27). When K=4,6, MDP exhibited the most pronounced increase in conflict number (average from 0.17∼0.57), approximately a fivefold growth. In contrast, our proposed method consistently maintained an average conflict number of 0.11. Figure 11 shows the conflict distribution of a 40×40 map involving 2∼10 humans. When K=2, no conflict occurs in our method, whereas t=4 in A*, t=3 in MDP, and t=24 in RRT. Particularly, when the number of humans doubled (K=8), A*, RRT and our method showed a twofold increase in conflict number compared to K=4, while MDP exhibited a nearly sixfold escalation. For all cases (*K*= 2∼10), the first conflict in our method occurs much later than the other compared methods.

The task success rate demonstrates significant differences: our method achieves 100% success at K=2 and declines to 70% at K=10, while A*, MDP, and RRT drop below 50% for K=8 and K=10. Notably, the reward metric showcases our method’s balanced optimization, delivering positive rewards (2.10∼1.26) throughout. By contrast, A*, MDP, and RRT yield negative values (ranging from 0.10∼−0.68 for A*, 0.34∼−2.58 for MDP, 0.28∼−1.10 for RRT) in high-density settings.

**Figure 12:**
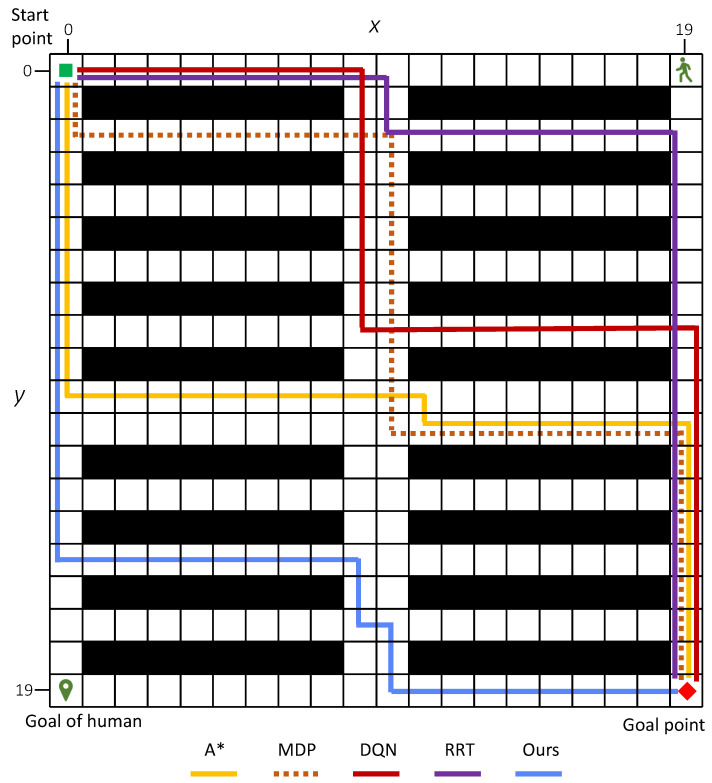
Examples of generated paths in 20 × 20 grip map with K=1.

**Figure 13:**
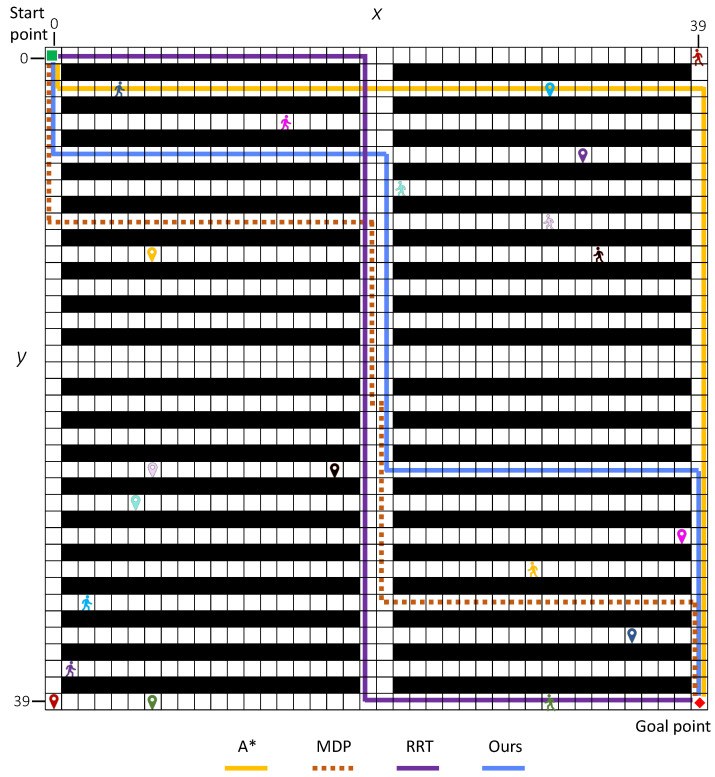
Examples of generated paths in 40 × 40 grip map with K=10.

In the most challenging scenario with human number K=10, our method reduces the average number of conflicts by −69.8%, −54.8%, and −73.4% compared with A*, MDP, and RRT, respectively $\left(\frac{0.42-1.39}{1.39}=-69.8\%,\;\frac{0.42-0.93}{0.93}=-54.8\%,\frac{0.42-1.60}{1.60}=-73.4\%\right)$. Moreover, our method significantly improves the average task success rate by 94.4% over A*, 70.7% over MDP, and 118.75% over RRT, respectively $\left(\frac{0.70-0.36}{0.36}=94.4\%,\;\frac{0.70-0.41}{0.41}=70.7\%,\frac{0.70-0.32}{0.32}=118.75\%\right)$. In terms of average reward, our method achieves a positive value of 1.26, which is substantially higher than those of A* (−0.68), MDP (−2.58), and RRT (−1.10). These simulation results clearly demonstrate that our method outperforms both A* and MDP across multiple metrics. For demonstration purposes, Figure 12 and Figure 13 display examples of the generated paths in maps with 20×20 and 40×40.

From the simulation results of the above three scenarios, we can see that the proposed method is more effective in solving potential conflicts at the planning level with stochastically moving humans. As discussed previously, our algorithm adopts a multi-path generator to generate multiple path candidates to find a safe path. Additionally, a redundant-path eliminator is proposed to reduce the candidate number while maintaining the diversity.

It should be noted that conflicts may still occur. Although the proposed algorithm effectively reduces the number of conflicts, there remains a gap toward our ultimate goal of completely resolving conflicts at the planning level within a single planning process. The algorithm is only tested in a warehouse grid environment with simple rectangle obstacles (shelves). The complex environment with different shapes of obstacles will be tested once the safe-path-planning problem is solved.

As discussed above, one main limitation of this work is that we cannot completely resolve all conflicts within a single planning process. Another limitation is that computational time has not been considered in this study. As described previously, the overall time computational complexity of the proposed method is $O\left(N|B\_list||A|+N\right)+O\left(N^{2}\right)+O\left(|\chi |T\right)\approx O\left(N^{2}\right)$. Currently, we concentrate on addressing human–robot conflicts at the planning level, while computational cost is not taken into account. We will improve the algorithm and enhance computational efficiency in our future work. In addition, taking the environment exploration with MDP into consideration, the computational cost remains relatively high, particularly in generating multiple paths using the RL-based method. As RL is effective in handling unknown environments, we employed it to generate multiple diverse paths despite its high computational cost. In addition, the human motion model adopted in this work is a simple stochastic one. We are currently developing an improved approach and will present the new algorithm in our future work.

## 5. Conclusions and Future Work

We address the problem of safe path planning in human-shared environments, where a mobile robot operates alongside stochastically moving humans. To tackle this challenge, we propose an RL-based safe path planner that generates optimal paths while accounting for stochastic, human-related risks at the planning level. The planner incorporates an MDP learner to explore the environment, on which a multi-path generator is built to produce multiple optimal and sub-optimal path candidates. To ensure path diversity while minimizing computational cost, a redundant path eliminator is introduced to filter out similar or unnecessary paths. By integrating stochastic human risk predictions from a human risk predictor, the decision unit module of the planner can generate an optimal and safe path for the robot in human-shared environments. Simulation results demonstrate the effectiveness of the proposed approach. In high-density settings (a 40×40 grid map with 10 humans), the proposed method reduces the average number of conflicts by −69.8%, −54.8%, and −73.4% compared with A*, MDP, and RRT methods, respectively. Meanwhile, it improves task success rates by 94.4%, 70.7%, and 118.75% relative to the same baseline methods.

Ongoing work includes the following: (1) extending the proposed planner from a single agent to multiple agents; (2) enhancing the risk prediction with CVaR; and (3) studying the theoretical safety guarantees of generated paths.

## Figures and Tables

**Table 1:** The result of 100 simulations with different learning rate α.

α=	0.1	0.2	0.3	0.4	0.5	0.6	0.7	0.8	0.9
Average conflict number	0.06	0.05	0.05	0.05	0.04	0.05	0.03	0.05	0.04
Average task success rate	0.95	0.95	0.95	0.95	0.96	0.95	0.97	0.95	0.97
Average reward	7.98	8.02	8.00	8.00	8.02	8.00	8.04	8.00	8.04

**Table 2:** The result of 100 simulations with different discount factor γ.

γ=	0.1	0.2	0.3	0.4	0.5	0.6	0.7	0.8	0.9
Average conflict number	0.03	0.03	0.03	0.05	0.03	0.03	0.05	0.03	0.02
Average task success rate	0.97	0.98	0.97	0.97	0.97	0.98	0.95	0.97	0.98
Average reward	8.04	8.04	8.04	8.00	8.04	8.04	8.00	8.04	8.06

**Table 3:** The result of 100 simulations with different exploration rate ϵ.

ϵ=	0.1	0.2	0.3	0.4	0.5	0.6	0.7	0.8	0.9
Average conflict number	0.06	0.02	0.03	0.06	0.04	0.05	0.02	0.03	0.03
Average task success rate	0.94	0.98	0.97	0.97	0.96	0.95	0.98	0.97	0.98
Average reward	7.98	8.06	8.04	7.98	8.03	8.00	8.06	8.04	8.06

**Table 4:** Simulation results on different size of maps. The numerical value represents the mean performance obtained from 100 rounds of simulation. The asterisk (*) denotes a statistically significant difference from the proposed method (p<0.05, Wilcoxon test), whereas the hyphen (-) indicates no significant difference (p≥0.05). The best results on each item are highlighted in bold font for each comparison.

Human	Grid Map	Methods	Average	Task	Reward	Cost Time (s)
**Number**			**Conflict Number**	**Success Rate**		
		A*	0.19 ± 0.48 (*)	0.85 ± 0.36 (*)	7.72 ± 0.97 (*)	<0.01
		MDP	0.09 ± 0.32 (-)	0.92 ± 0.27 (-)	7.91 ± 0.64 (-)	9.09
K=1	10×10	DQN	0.05 ± 0.22 (-)	0.94 ± 0.24 (-)	8.00 ± 0.44 (-)	80.62
		RRT	0.19 ± 0.48 (*)	0.84 ± 0.37 (*)	7.72 ± 0.97 (*)	<0.01
		Ours	**0.03 ± 0.17**	**0.97 ± 0.17**	**8.04 ± 0.34**	11.19
		A*	0.10 ± 0.46 (*)	0.93 ± 0.26 (*)	5.90 ± 0.92 (*)	<0.01
		MDP	0.05 ± 0.22 (-)	0.94 ± 0.24 (-)	5.89 ± 1.09 (*)	23.40
K=1	20×20	DQN	0.17 ± 0.45 (*)	0.65 ± 0.48 (*)	2.16 ± 5.44 (*)	1393.16
		RRT	0.27 ± 0.99 (*)	0.88 ± 0.32 (*)	5.56 ± 1.97 (*)	<0.01
		Ours	**0.01 ± 0.10**	**0.99 ± 0.10**	**6.08 ± 0.20**	25.41
		A*	0.06 ± 0.24 (*)	0.94 ± 0.24 (*)	1.98 ± 0.47 (-)	<0.01
		MDP	0.07 ± 0.26 (*)	0.93 ± 0.26 (*)	1.96 ± 0.51 (*)	58.32
K=1	40×40	DQN	0.29 ± 0.52 (*)	0.27 ± 0.44 (*)	−5.52 ± 5.15 (*)	4040.98
		RRT	0.13 ± 0.44 (*)	0.89 ± 0.31 (*)	1.72 ± 1.47 (*)	<0.01
		Ours	**0.00 ± 0.00**	**1.00 ± 0.00**	**2.10 ± 0.04**	57.21

**Table 5:** Average results under the environmental setting of a 40×40 grid map and 2∼10 human. The numerical value represents the mean performance obtained from 100 rounds of simulation. The asterisk (*) denotes a statistically significant difference from the proposed method (p<0.05, Wilcoxon test), whereas the hyphen (-) indicates no significant difference (p≥0.05). The best results on each item are highlighted in bold font for each comparison.

Human Number	Methods	Average Conflict Number	Task Success Rate	Reward	Cost Time (s)
K=2	A*	0.12 ± 0.38 (*)	0.90 ± 0.30 (*)	1.86 ± 0.76 (*)	<0.01
	MDP	0.14 ± 0.51 (*)	0.86 ± 0.35 (*)	1.38 ± 2.19 (*)	69.15
	RRT	0.20 ± 0.57 (*)	0.86 ± 0.35 (*)	1.72 ± 1.47 (*)	<0.01
	Ours	**0.00 ± 0.00**	**1.00 ± 0.00**	**2.10 ± 0.04**	59.40
K=4	A*	0.48 ± 1.21 (*)	0.71 ± 0.45 (*)	1.14 ± 2.42 (*)	<0.01
	MDP	0.17 ± 0.38 (-)	0.75 ± 0.43 (*)	0.69 ± 3.15 (*)	116.63
	RRT	0.66 ± 1.27 (*)	0.60 ± 0.49 (*)	0.78 ± 2.54 (*)	<0.01
	Ours	**0.11 ± 0.53**	**0.94 ± 0.24**	**1.88 ± 1.05**	63.60
K=6	A*	0.57 ± 0.79 (*)	0.59 ± 0.49 (*)	0.96 ± 1.58 (*)	<0.01
	MDP	0.57 ± 1.35 (*)	0.65 ± 0.48 (*)	0.34 ± 3.60 (*)	156.75
	RRT	0.85 ± 1.51 (*)	0.56 ± 0.50 (*)	0.28 ± 3.18 (*)	<0.01
	Ours	**0.11 ± 0.42**	**0.92 ± 0.27**	**1.88 ± 0.84**	69.20
K=8	A*	1.00 ± 1.62 (*)	0.44 ± 0.50 (*)	0.10 ± 3.25 (*)	<0.01
	MDP	0.66 ± 1.20 (*)	0.48 ± 0.50 (*)	−1.83 ± 5.22 (*)	197.78
	RRT	1.34 ± 2.73 (*)	0.49 ± 0.50 (*)	−0.58 ± 5.46 (*)	<0.01
	Ours	**0.21 ± 0.62**	**0.84 ± 0.37**	**1.68 ± 1.24**	73.40
K=10	A*	1.39 ± 1.67 (*)	0.36 ± 0.48 (*)	−0.68 ± 3.33 (*)	<0.01
	MDP	0.93 ± 1.47 (*)	0.41 ± 0.49 (*)	−2.58 ± 5.25 (*)	234.36
	RRT	1.60 ± 2.54 (*)	0.32 ± 0.47 (*)	−1.10 ± 5.08 (*)	<0.01
	Ours	**0.42 ± 0.78**	**0.70 ± 0.46**	**1.26 ± 1.55**	80.01

## Data Availability

The data generated by the proposed algorithm are shown in the manuscript and no additional data is used. The original code presented in the study are openly available in GItHub at https://github.com/GitAB2/Path_planning_mprl (accessed on 29 September 2025).
